# Association of tuberculosis treatment with pregnancy complications: A protocol for systematic review and meta-analysis

**DOI:** 10.1097/MD.0000000000027849

**Published:** 2021-11-19

**Authors:** Moloko A. Kgathi, Wendy N. Phoswa

**Affiliations:** Department of Life and Consumer Sciences, University of South Africa (UNISA), Science Campus, Private Bag X6, Florida, Roodepoort, South Africa.

**Keywords:** preeclampsia, tuberculosis, tuberculosis treatment

## Abstract

**Background:**

The increasing burden of tuberculosis (TB) remains a very serious concern around the world, and account for a decreased quantity and quality of life. However, there is a limited epidemiology of the association of TB treatment with pregnancy. We aim to assess the effects of TB treatment in pregnancy complications.

**Methods:**

This will be a systematic review and meta-analysis of published studies on the association of TB treatment with pregnancy, retrieved from ScienceDirect, Web of Science, LILACS, Pubmed, Google scholar, Embase, Medline, ResearchGate, EBSCOhost and Cochrane library databases. The eligibility of the studies will be screened in accordance to the selection criteria by two independent reviewers. The quality and risk of bias of eligible studies will be performed by both reviewers using the Hoy tool and Grading of Recommendations Assessment, Development and Evaluation (GRADE) tool in accordance to the measured outcomes (Hypertension in pregnancy, Pre-eclampsia, Hypertensive disorders of pregnancy, Fetal growth restriction, Miscarriage and Recurrent spontaneous abortion). A data charting table will be used to extract background information and process the data items from each eligible study. The data will be analysed using Review Manager 5.3 (RevMan 5.3) software. Generic Inverse Variance method will be used for meta-analysis of both, individually and cluster randomized trials.

**Ethics and dissemination:**

The review and meta-analysis will not require ethical approval and the findings will be published in peer-reviewed journals and presented at local and international conferences. In addition, the study findings will be made accessible to the national committee of TB to formulate TB guidelines for their respective settings.

**Systematic review registration:**

International prospective Register of Systematic Reviews (PROSERO) number: CRD42021231872.

## 1 Introduction

The burden of tuberculosis (TB) remains a very serious concern around the world.^[1]^ Falana et al mentioned that globally, TB emerges to account for decreased quantity and quality life.^[2]^ According to Esmail et al the World Health Organisation estimated that about 2 million people have died from TB in the same year. The report is said to have characterized tuberculosis as the most infectious disease and the most leading cause of death around the globe.^[3]^ According to Van der Walt et al in 2018, South Africa was estimated to have had about 11 000 TB drug-resistant cases, of which 62% were estimated to be of Multidrug-resistant TB (MDR-TB). Van der Walt et al elaborated that the 62% estimate is driven by “Human Immunodeficiency Virus epidemic”.^[4]^

TB can occur as latent TB infection, TB disease or HIV-related TB disease.^[5]^ The World Health Organisation (WHO) mentioned that TB treatment during pregnancy differs due to immunological alterations induced by pregnancy. Therefore, latent TB infection can be treated using “Rifampin (RIF) (4R), Isoniazid (INH) and RIF (3HR), INH (6H/9H) with pyridoxine (Vitamin B) supplementation or INH and Rifapentine (3HP)” regime. However, INH and Rifapentine (3HP) is not recommended for pregnant women nor women expecting to conceive during TB treatment period due to lack of sufficient research on its safety during pregnancy.^[6]^ According to WHO guidelines TB disease can be treated using INH, Rifampin (RIF) and ethambutol (EMB), Streptomycin and Pyrazinamide (PZA). However, Streptomycin and PZA are not recommended because Streptomycin is mentioned to have shown harmful effects on the fetus whereas PZA's effects is not clearly defined. WHO further suggested HIV-related TB disease during pregnancy should be treated similar to a non-HIV related TB disease.^[6]^

Van der Walt et al stated that pregnant women are often excluded during clinical trials, therefore, there exist no sufficient data to use of DR-TB treatment for pregnant woman.^[4]^ Thus, treatment of drug-resistant TB (DR-TB) is still a challenge. Moro et al mentioned that the current recommended treatment is Isoniaze for nine months without delay.^[7]^ However, the updated WHO guidelines of (2016) suggests that drugs such as bedaquiline, delamanid, linezolid and fluoroquinolones are some of the most recommended drugs for DR-TB therapy.^[5]^

TB pregnant women have a substantial burden, which have an estimated prevalence amongst pregnant and post-partum women starting from 0.06% to 7.2%. These estimates can reach as high as 11%. Bekker et al mentioned that HIV-infected pregnant woman were having a prevalence of 5.67 times more with a likelihood to have extrapulmonary TB.^[8]^ Therefore, HIV-co-infected pregnant woman are at risk of TB infection with TB prevalence of 1–11% in comparison to 0.06–0.53% of HIV-negative pregnant woman.^[8]^

TB positive pregnant woman tend to be more vulnerable, even during a post-partum period.^[9]^ During pregnancy, T-helper (Th-1/ Th-2) cells have showed to be reduced, and these predominantly increases susceptibility rate to new infections. Therefore, this contributes to TB reactivation.^[3,10]^ During early stages of post-partum period, Th-1 cells suppression may be reversed. This reversal might be due to the exacerbation of symptoms akin to the immune reconstitution syndrome.^[3]^ Research has however showed that tuberculosis during pregnancy can also be related to poor outcomes such as “perinatal death, stillbirths, premature birth, low birth weight (LBW), an increased risk of preterm birth, intrauterine growth restriction and hyaline membrane disease”.^[8,10]^ TB is mentioned to be the most common cause of maternal deaths amounting to more than 35% of all maternal deaths. However, maternal death is mentioned to occur mostly in HIV-co-infected pregnant women.^[8]^ Pregnancy complications of exposure to TB treatment is not well known, however TB treatment in HIV co-infected and non-HIV co-infected pregnant woman yields a positive result. The study by Denti et al mentioned that pregnancy triggers physiological changes that has a major impact on the “absorption, distribution, renal or hepatic clearance and metabolism” of TB drugs during therapy. Findings from this study further mentioned that Rifampin is the major sterilizing drug in the therapy regime of drug sensitive TB and Azithromycin the precursor of pregnancy-induced cholestasis.^[11]^ Research showed that Isoniazid TB therapy during pregnancy had the ability to induce congenital abnormalities known as “talipes equinovarus” in an infant.^[12]^

Esmail et al advised that drug-resistant TB treatment and its construction regimen during pregnancy should be in accordance to gestational age of the foetus and teratogenic anti-TB treatment effects should be weighed.^[3]^ However, due to teratogenic conditions, treatment can often be delayed to second trimester. Furthermore, Beck–Friis et al mentioned that there are only few studies conducted on tuberculosis treatment during pregnancy and pregnant women often experiences severe hepatotoxicity unlike non-pregnant woman.^[13]^ Therefor it is of paramount significant to closely monitor drug-resistant TB treatment in pregnancy to evade complications. In support to Esmail et al, Beck–Friis et al mentioned that liver transaminases must be monitored carefully during the treatment of TB in pregnant woman.^[3,13]^ Furthermore, increase in enzymes of the liver must be observed and temporary withdrawal of drugs must be considered if there is an increase of enzymes.^[10]^ In these regard, the main objective of this review is to investigate the association of TB drugs among TB positive pregnant women.^[7]^

## 2 Research question

What might be the association between TB treatment and pregnancy complications?

## 3 Research objectives

1. To assess the effects of TB treatment in pregnant woman.
2. To assess the association of TB with pregnancy
3. To examine TB treatment complications in pregnant women.
4. To investigate the association of TB drugs among TB positive pregnant women.

## 4 Methods

This will be a systematic review and meta-analysis of published studies. This protocol is written in line with the recommendations of the Preferred Reporting Items for Systematic Reviews and Meta-analysis for Protocols (PRISMA-P) guidelines 2015.^[14]^ The results will be reported based on the PRISMA 2015,^[14]^ statement and article screening and selection process will also be demonstrated through a PRISMA-P flow diagram. Furthermore, the current protocol has been registered with the International Prospective Register of Systematic Reviews (PROSPERO): CRD42021231872.

## 5 Eligibility

### 5.1 Study design

This systematic review and meta-analysis will include randomized control trials, cohort, and matched cohort with a clearly defined population and interventions used. While, observational studies, reviews, case studies, and animal studies will be excluded in this study.

### 5.2 Participants

TB positive pregnant women.

### 5.3 Intervention

Tuberculosis treatment.

### 5.4 Comparator

TB negative pregnant women.

### 5.5 Outcomes

Hypertension in pregnancy, hypertensive disorders of pregnancy, pre-eclampsia, fetal growth restriction, miscarriage, recurrent spontaneous abortion.

### 5.6 Inclusions criteria

1. Evidence published in the English language.
2. Evidence published between the years 2010–2020.
3. Evidence from published global Randomized control trial, matched cohort, and cohort studies of TB treatment related pregnancy outcomes/ complication.
4. All of the criteria defining the impact of TB treatment on the incidence of adverse pregnancy and foetal outcomes.

### 5.7 Exclusion criteria

1. Study which does not have the outcomes of interest as objectives.
2. Case reports, expert opinions and review/meta-analysis.
3. Evidence published before the year 2010.
4. Evidence from TB non-pregnant women will be excluded because the impact of antidepressants is expected to be evaluated in pregnant women.

### 5.8 Search strategy

The following databases will be searched for eligible studies: Science direct, Medline, Embase, Pubmed, Africa Wide, Google scholar, ResearchGate, EBSCOhost, Web of Science, and the Cochrane Library, and LILACS. Medical subject headings (MeSH) such as “(((((((((((((TB[All Fields] AND positive[All Fields] AND (“pregnant women”[MeSH Terms] OR (“pregnant”[All Fields] AND “women”[All Fields]) OR “pregnant women”[All Fields])) AND ((“tuberculosis”[MeSH Terms] OR “tuberculosis”[All Fields]) AND positive[All Fields] AND (“pregnant women”[MeSH Terms] OR (“pregnant”[All Fields] AND “women”[All Fields]) OR “pregnant women”[All Fields]))) AND (TB[All Fields] AND (“therapy”[Subheading] OR “therapy”[All Fields] OR “treatment”[All Fields] OR “therapeutics”[MeSH Terms] OR “therapeutics”[All Fields]))) AND (TB[All Fields] AND negative[All Fields] AND (“pregnant women”[MeSH Terms] OR (“pregnant”[All Fields] AND “women”[All Fields]) OR “pregnant women”[All Fields]))) AND (“hypertension, pregnancy-induced”[MeSH Terms] OR (“hypertension”[All Fields] AND “pregnancy-induced”[All Fields]) OR “pregnancy-induced hypertension”[All Fields] OR (“hypertension”[All Fields] AND “pregnancy”[All Fields]) OR “hypertension in pregnancy”[All Fields])) OR (“abortion, spontaneous”[MeSH Terms] OR (“abortion”[All Fields] AND “spontaneous”[All Fields]) OR “spontaneous abortion”[All Fields] OR “miscarriage”[All Fields])) OR (hypertensive[All Fields] AND (“disease”[MeSH Terms] OR “disease”[All Fields] OR “disorders”[All Fields]) AND (“pregnancy”[MeSH Terms] OR “pregnancy”[All Fields]))) OR (“pre-eclampsia”[MeSH Terms] OR “pre-eclampsia”[All Fields] OR (“pre”[All Fields] AND “eclampsia”[All Fields]) OR “pre eclampsia”[All Fields])) OR (“fetal growth retardation”[MeSH Terms] OR (“fetal”[All Fields] AND “growth”[All Fields] AND “retardation”[All Fields]) OR “fetal growth retardation”[All Fields] OR (“fetal”[All Fields] AND “growth”[All Fields] AND “restriction”[All Fields]) OR “fetal growth restriction”[All Fields])) OR (“abortion, spontaneous”[MeSH Terms] OR (“abortion”[All Fields] AND “spontaneous”[All Fields]) OR “spontaneous abortion”[All Fields] OR “miscarriage”[All Fields])) OR (recurrent[All Fields] AND (“abortion, spontaneous”[MeSH Terms] OR (“abortion”[All Fields] AND “spontaneous”[All Fields]) OR “spontaneous abortion”[All Fields] OR (“spontaneous”[All Fields] AND “abortion”[All Fields])))) AND ((“random allocation”[MeSH Terms] OR (“random”[All Fields] AND “allocation”[All Fields]) OR “random allocation”[All Fields] OR “randomized”[All Fields]) AND (“prevention and control”[Subheading] OR (“prevention”[All Fields] AND “control”[All Fields]) OR “prevention and control”[All Fields] OR “control”[All Fields] OR “control groups”[MeSH Terms] OR (“control”[All Fields] AND “groups”[All Fields]) OR “control groups”[All Fields]) AND (“Trials”[Journal] OR “trials”[All Fields]))) OR (“cohort studies”[MeSH Terms] OR (“cohort”[All Fields] AND “studies”[All Fields]) OR “cohort studies”[All Fields] OR “cohort”[All Fields])) OR (matched[All Fields] AND (“cohort studies”[MeSH Terms] OR (“cohort”[All Fields] AND “studies”[All Fields]) OR “cohort studies”[All Fields] OR “cohort”[All Fields])) and free text searches will be used to search the eligible articles which will be saved to the citation manager Zotero v5.0.81 (Zotero.org). This software will also be used to remove duplicates. The title and abstracts of the articles remaining after exclusion of duplicates will be assessed for eligibility according to the inclusion and exclusion criteria.

### 5.9 Study selection

The full text of all potentially eligible studies will then be reviewed by two independent reviewers (MAK and WNP), and any disagreement between reviewers with respect to eligible studies for inclusion in the analysis will be assessed for more eligible studies. Initially, studies will be screened by the titles, abstracts, keywords, and synonyms then followed by the identification of the full-text articles. Should discrepancies arise between 2 authors (MAK, WNP), a third author will screen such studies, and consensus will be reached through discussion. Zotero v5.0.81 (Zotero.org) will be used to manage extracted data items, including saving relevant and excluded studies with reasons. Importantly, reference lists of included studies will be screened to confirm that no relevant studies are left out. Studies meeting the inclusion criteria will then be subjected to data collection, critical appraisal, risk, and quality evaluation.

## 6 Data management

### 6.1 Data collection process

The reviewers (MAK and WNP) will develop a data extraction form that will be used in the collection relevant data items. To reduce data entry errors, selected studies will be independently assessed by two reviewers (MAK and WNP), the third reviewer will be consulted for arbitration in case of any disagreements.

### 6.2 Data items

Extracted data items will include the author's name, year of publication, HIV status: (report percentages + and -), TB status: (report percentages + and -), HIV treatment, TB treatment, gestational age, adverse maternal outcomes: Hypertensive disorders of pregnancy, pregnancy induced hypertension, pre-clampsia, adverse fetal outcomes: Fetal growth restriction, miscarriage, recurrent spontaneous abortion.

### 6.3 Risk of bias in individual studies

To evaluate the potential risk of bias in randomized control trials, cohort, and matched cohort, Cochrane collaboration tool for assessing bias^[15]^ and Downs and Black checklist^[16]^ will be used. Two independent reviewers (MAK and WNP) will appraise all included studies and a third reviewer will be consulted in cases of disagreements.

### 6.4 Data synthesis

A summary of findings table will be used to provide a synthesis of the main outcomes of included studies. data will be analyzed with Rev Manager (Version 5.3) to conduct a meta-analysis. To measure statistical heterogeneity between studies, I^2^ and Chi squared statistical tests will be used.^[17,18]^ An I^2^ value of > 50% will be considered substantial heterogeneity.^[19]^ To find the sources of heterogeneity within the included studies, a subgroup analysis and meta-regression comparing the study estimates from different study-level characteristics, quality, intervention type, and the reported effect measure of adverse events will be conducted.

## 7 Quality assessment of the cumulative evidence

The Grading of Recommendations, Assessment, Development and Evaluation assessment tool^[20]^ will be used to assess the overall quality of evidence. Moreover, the quality of each included study will be independently evaluated by 2 authors (MAK, WNP). The third author will adjudicate in cases of disagreements. The quality of evidence will be assessed based on several factors such as study limitations, indirectness of results, and publication or reporting bias. The evidence of each outcome will be rated as high, moderate, low, or very low.

## 8 Discussion

Tuberculosis (TB) infection during pregnancy has been reported to be associated with maternal and fetal adverse effects and has not been critically elucidated.^[21]^ Therefore, this systemic review and meta-analysis aimed to investigate the effect of TB treatment on pregnancy complications. Findings from this study will help to give a better understanding on the pathophysiology of TB in pregnancy complications as wells as to understand the effect of TB treatment in pregnancy complications in order to improve future diagnostic criteria's, thereby reducing maternal and fetal adverse outcomes.

## Author contributions

**Conceptualization:** Wendy Phoswa, Moloko Adolph Kgathi.

**Investigation:** Wendy Phoswa, Moloko Adolph Kgathi.

**Methodology:** Wendy Phoswa.

**Resources:** Wendy Phoswa.

**Software:** Wendy Phoswa.

**Supervision:** Wendy Phoswa.

**Validation:** Wendy Phoswa, Moloko Adolph Kgathi.

**Visualization:** Wendy Phoswa.

**Writing – original draft:** Wendy Phoswa, Moloko Adolph Kgathi.

**Writing – review & editing:** Wendy Phoswa, Moloko Adolph Kgathi.
